# Antimicrobial Susceptibility Patterns and Biofilm Formation of *Staphylococcus aureus* Strains Isolated from Pediatric Patients with Atopic Dermatitis

**DOI:** 10.3390/microorganisms14020311

**Published:** 2026-01-29

**Authors:** Carolina Romo-González, Alejandra Aquino-Andrade, Abril Pérez-Carranza, Diana Chaparro-Camacho, Andrea Becerril-Osnaya, Maria Teresa García-Romero

**Affiliations:** 1 Experimental Bacteriology Laboratory, Instituto Nacional de Pediatría, Mexico City 04530, Mexico; cromog@pediatria.gob.mx; 2 Molecular Microbiology Laboratory, Instituto Nacional de Pediatría, Mexico City 04530, Mexico; aaquinoa@pediatria.gob.mx; 3 Diagnostic Bacteriology, Facultad de Estudios Superiores Cuautitlán, Universidad Nacional Autónoma de México (UNAM), Cuautitlán Izcalli 54740, Mexico; 4 Department of Dermatology, Instituto Nacional de Pediatría, Mexico City 04530, Mexico

**Keywords:** *S. aureus*, antimicrobial susceptibility, biofilm, children, atopic dermatitis

## Abstract

Atopic dermatitis (AD) is a chronic inflammatory skin disease characterized by barrier dysfunction and susceptibility to *Staphylococcus aureus* colonization. Biofilm formation modifies antibiotic resistance and the host immune response. This longitudinal study analyzed antimicrobial susceptibility and biofilm formation in 136 *S. aureus* isolates obtained over 18 months from lesional, nonlesional, and nasal samples of 26 pediatric patients with moderate-to-severe AD. Antimicrobial susceptibility testing was determined by the disk diffusion method, and biofilm production was quantified using a crystal violet microtiter assay. Clinical parameters, including disease severity, treatment response, and the administration of dilute bleach baths, were evaluated in relation to bacterial characteristics. Overall, 60.2% of isolates exhibited moderate-to-strong biofilm production, significantly associated with severe AD at baseline (*p* = 0.01), lack of clinical improvement (*p* = 0.04), and persistent moderate-to-severe disease (*p* = 0.01). Resistance rates for penicillin, gentamicin, clindamycin, and erythromycin exceeded 15%. Isolates from patients using dilute bleach baths showed greater resistance to ciprofloxacin (*p* < 0.0001) and exhibited constitutive or inducible macrolide–lincosamide–streptogramin B (MLS_B_) resistance, with *erm*A detected in 80% of inducible cases. In conclusion, *S. aureus* biofilm formation is linked to disease severity and treatment failure in pediatric AD, underscoring the importance of culture-guided, targeted therapeutic strategies.

## 1. Introduction

Atopic dermatitis (AD) is a chronic multifactorial inflammatory skin disease characterized by alterations in epidermal barrier function and an exaggerated innate immune response. It affects 20–30% of children and is among of the most frequent reasons for medical consultation [1,2]. Manifestations of AD include erythematous, scaly patches that are intensely pruritic, leading to a vicious cycle of scratching that exacerbates the disease. Patients frequently exhibit colonization or secondary infection of AD by *S. aureus*, and this pathogen plays a role in the physiopathology of the disease by producing superantigens, decreasing the expression of antimicrobial peptides, and forming biofilms that impair the effects of targeted treatment [1,2].

The antimicrobial susceptibility of *S. aureus* strains that cause disease or are isolated from patients with AD has been reported to differ from that of strains isolated from healthy controls. Since distinguishing mild secondary infections from disease flares can be challenging, antibiotics are often used as empiric treatment [3]. Furthermore, resistance to antibiotics recommended as first-line empirical therapy for skin infections may exceed the optimal threshold (prevalence of >15% resistant isolates) [4,5,6,7,8,9], and methicillin resistance and multidrug resistance are potentially associated with age, severity of AD, and treatment [6,10]. *S. aureus* produces biofilms, which protect bacteria from environmental hazards, innate immune response-derived antimicrobial peptides (AMPs), antibiotics, and phagocytosis, and contribute to the pathogenesis of chronic infections. Biofilms have been detected on the skin of AD patients and possibly contribute to the inflammation process [11,12,13,14]. In this study, we sought to analyze the antimicrobial susceptibility profiles, characterize biofilm formation, and identify differences associated with clinical characteristics in *S. aureus* strains isolated longitudinally from children with AD.

## 2. Materials and Methods

This longitudinal observational study was performed at the National Institute of Pediatrics (NIP) (IRB approval number 073/2019). We obtained *S. aureus* isolates from a cohort of children with moderate–severe AD who attended the dermatology clinic from July 2017 to December 2018. We procured informed consent from all subjects involved in the study. The severity of AD was quantified via the Severity Score for AD (SCORAD) [15]. Superficial swabs were obtained from 7 body sites (nares, antecubital folds, and popliteal folds) in up to 5 visits (one baseline and four follow-ups). Cultures and colony isolation were performed at the Experimental Bacteriology Laboratory of the NIP. For each patient, we selected up to 3 colonies: one isolate from a lesional site, one from a non-lesional site, and one from the nares, at baseline and at a follow-up visit (1 to 4 months after). Isolates obtained during initial visits were given preference over those from the latest visit. For each isolate, we (1) performed antimicrobial susceptibility testing via the disk diffusion test, (2) analyzed in vitro biofilm production, and (3) performed detection of the *erm*A, *erm*B, *erm*C, and *msr*A genes by PCR. Patients were categorized as those receiving standard treatment for their AD (medium- to high-potency topical steroids and/or calcineurin inhibitors) and those receiving adjunctive dilute bleach baths (0.006%).

### 2.1. Sample Collection and Processing

Each sample was then streaked onto the surface of sheep blood agar and mannitol salt agar (Becton Dickinson, Le Pont de Claix, France) plates and incubated at 37 °C for 24 h for purity verification and identification. After the incubation period, colonies were identified by mannitol fermentation and colony morphology, in addition to Gram staining, and the final identification was performed using conventional biochemical tests (e.g., catalase, coagulase, and DNase); subsequently, all isolates included in this study were sequenced according to a previous study [16]. For this study, a total of 136 *S. aureus* isolates from patients with AD were selected. All the isolates were further tested for antimicrobial susceptibility and biofilm formation.

### 2.2. Patients’ Clinical Characteristics

We investigated differences in the antimicrobial susceptibility patterns and biofilm formation *of S. aureus* isolates according to the following categories based on the clinical characteristics of the patients:

1. Body sites from which isolates were obtained: affected skin, unaffected skin, or nares;
2. Severity of AD at baseline: moderate or severe;
3. Baseline visit or follow-up visit (once patients had received treatment);
4. Number of body sites colonized by *S. aureus* at baseline: ≤three sites or more than three sites;
5. Adjunctive treatment with or without dilute bleach baths;
6. Response to treatment (decrease of at least 15 SCORAD points from baseline to final visit);
7. Persistent moderate-to-severe AD throughout all visits.

### 2.3. Antimicrobial Susceptibility Testing

The antimicrobial susceptibility profiles and the inducible clindamycin resistance (ICR) test were performed according to the guidelines of the Clinical and Laboratory Standards Institute (CLSI) [17,18]. A disk diffusion test was performed for penicillin (P), cefoxitin (FOX), erythromycin (E), clindamycin (CC), ciprofloxacin (CIP), tetracycline (TE), gentamicin (GM), trimethoprim-sulfamethoxazole (SXT), and linezolid (LIN) (Becton Dickinson, Franklin Lakes, NJ, USA). The concentrations of all antibiotic disks used in this study are detailed in Supplementary Table S1. The inducible macrolide–lincosamide–streptogramin B (iMLS_B_) was considered when the isolates were resistant to E or sensitive or intermediate to CC, with a positive inducible clindamycin resistance (ICR) test; the macrolide-streptogramin B (MS_B_) phenotype was considered when the ICR test was negative; and the constitutive phenotype (cMLS_B_) was considered if they were resistant to E and CC [19].

### 2.4. Biofilm Assay

Each isolate was grown on brain heart infusion (BHI) agar plates at 37 °C for 24 h. Next, the cell density was adjusted to 0.5 MacFarland (1.5 × 10^8^ CFU/mL) in 0.9% physiological saline solution. The strain controls for this test were the same as those used by Singh et al. [20]. Biofilm production was performed with BHI supplemented with 222.2 mM glucose, 116.9 mM saccharose, and 1000 mM NaCl. Overnight cultures were enriched with BHI, a suspension equivalent to 0.5 MacFarland was diluted 1:100 in freshly prepared BHI. Aliquots (100 μL) were transferred to 96-well microtiter plates. Sterile broth without culture served as a control. The plates were incubated at 37 °C for 48 h. After incubation, the contents of each well were gently decanted. The wells were washed 2–3 times with 200 μL of phosphate-buffered saline (PBS) to remove planktonic bacteria. Then, the plate was air-dried at room temperature and stained with 0.5% crystal violet. The wells were subsequently washed with distilled water 5–6 times to remove excess stain. The stain adherent to the walls was dissolved in 100 μL of alcohol/acetone (80:20), and the optical density was read at 570 nm via an Epoch Microplate Absorbance Reader (Agilent, Santa Clara, CA, USA). All experiments with clinical isolates were performed in triplicate, and mean OD values and standard deviations were calculated. The biofilms formed by the *S. aureus* isolates were classified as Non–biofilm producers (OD ≤ ODc); Weak biofilm producers (ODc < OD ≤ 2 × ODc); Moderate biofilm producers (2 × ODc < OD ≤ 4 × ODc); Strong biofilm producers (4 × ODc < OD) as suggested by Singh et al. [20].

### 2.5. Detection of ermA, ermB, ermC, and msrA Genes

DNA was obtained using the QIAmp^®^ DNA mini kit (Qiagen, Hilden, North Rhine–Westphalia, Germany). The DNA was eluted and stored at −20 °C until use. The genes *erm*A, *erm*B, *erm*C, and *msr*A were amplified by PCR using previously reported primers and conditions [21,22]. The primer sequences and amplification conditions are provided in the Supplementary Materials (Table S2). Positive control strains of *S. aureus* O2 (*erm*A and *erm*C) and O46 (*erm*A), previously sequenced and reported by Aguilar-Gómez et al. [23], were used as positive controls.

### 2.6. Statistical Analysis

Descriptive statistics were used to summarize continuous variables with means and standard deviations and categorical variables with numbers and percentages. Differences between groups were analyzed via the chi-square test or Fisher’s exact test (SPSS Version 21.0, IBM, Armonk, NY, USA). A *p*-value of ≤0.05 was considered significant.

## 3. Results

### 3.1. Characteristics of the S. aureus Isolates

We included 136 *S. aureus* isolates from 26 patients (Table 1). Fifty-five (40.4%) of the isolates were obtained from patients’ nares, 50 (36.7%) from affected skin, and 31 (22.7%) from unaffected skin. All patients had moderate or severe AD at baseline (SCORAD ≥ 25). At the baseline visit, all patients were prescribed emollients and moderate-potency topical steroids twice daily; 12 received adjunctive diluted bleach baths (0.006%) in addition to standard treatment, while 14 did not. Seventy-two isolates of *S. aureus* (52.9%) were obtained from the baseline visits. At the initial visit, 15 patients had >3 body sites colonized by *S. aureus*, and 58 isolates (80.5%) were obtained from these patients.

Sixty-four isolates (47%) were obtained from follow-up visits. Of these, 23 (35.9%) were isolated from 7 patients who received diluted bleach baths as adjunctive treatment, and 41 (64%) were isolated from 9 patients who did not. Most patients (18, 69.2%) responded well to treatment, and their disease severity decreased 15 or more SCORAD points from baseline to the end of the study; 96 isolates (70.5%) were derived from these individuals. Five patients (19.2%) had persistent moderate–severe AD throughout the study period, accounting for 58 isolates (42.6%).

### 3.2. Antimicrobial Susceptibility Patterns and Biofilm Production

In general, isolates of *S.* *aureus* had suboptimal resistance rates (>15%) to P (103, 75.7%), GM (49, 36%), CC (34, 25%), and E (34, 25%) (Table 2). The inhibition zone diameters, are provided together in Supplementary Table S1. All 136 isolates were methicillin sensitive. The MLS_B_ phenotype was documented in 58 (23.8%) of the isolates: 14 (10.2%) had cMLS_B_, 20 (14.7%) had iMLS_B_, and 1 (0.7%) had the MS_B_ phenotype (Table 3). The *erm*A gene was detected in 16 isolates (80%) with the iMLS_B_ phenotype, of which one was associated with *msr*A; *erm*B and *erm*C were not detected. In total, 23 (16.9%) isolates did not form biofilms, 31 (22.7%) were weak producers, 55 (40.4%) were moderate producers, and 27 (19.8%) were strong producers. Mean optical density (OD) values and standard deviations for isolates classified in different categories of biofilm producers are shown in Table S3.

We searched for differences between 2 categories of biofilm production (none or weak vs. moderate or strong producers) and antimicrobial susceptibility profiles, finding no difference. We further studied MLS_B_ resistance patterns and moderate or strong biofilm production in the isolates and found no differences (Table 3).

### 3.3. Clinical Characteristics, Antimicrobial Susceptibility Patterns, and Biofilm Formation

We analyzed the clinical characteristics of patients from whom the isolates were obtained, and the characteristics of the *S. aureus* isolates (Table 4). Patients with non-severe AD at baseline were more likely to have isolates from the initial visit that were resistant to P (*p* = 0.02). Those with severe AD at baseline were more likely to have *S. aureus* isolates that produced biofilms at moderate to strong levels (*p* = 0.01). Isolates from patients whose SCORAD improved throughout the study were more frequently resistant to P (*p* = 0.001) and predominantly classified as non-biofilm producers or weak biofilm producers (*p* = 0.04). Isolates from those whose SCORAD did not improve accordingly were more frequently resistant to CIP (*p* < 0.0001), GM (*p* = 0.03), CC (*p* = 0.01), and E (*p* = 0.02).

These isolates were also more likely to have cMLS_B_ and iMLS_B_ (*p* < 0.0001). *S. aureus* isolates from patients with persistent moderate–severe AD throughout the study were significantly more frequently resistant (or intermediately resistant) to CIP (*p* = 0.03) but sensitive to P (*p* < 0.0001) and TE (*p* = 0.02). These isolates were also more likely to have cMLS_B_ and iMLS_B_ (*p* < 0.0001) and be moderate-strong biofilm producers (*p* = 0.01). Isolates collected during follow-up visits from patients who did not receive dilute bleach baths as adjunctive treatment were more likely to be resistant to CIP (*p* < 0.0001). These isolates were also more likely to have cMLS_B_ and iMLS_B_ (*p* = 0.01). Isolates from patients who received adjunctive treatment with dilute bleach baths were more likely to be resistant to P (*p* = 0.004).

## 4. Discussion

In this study of 136 isolates of *S. aureus* from children with AD, we found a predominance of moderate to strong biofilm production capacity (60.2%), which was associated with clinical characteristics such as disease severity at baseline, lack of response to treatment, and persistent moderate–severe AD throughout the study visits. We also found suboptimal antimicrobial susceptibility rates (<15%) to CC, GM, P, and E. According to proposed recommendations, when the prevalence of methicillin- and clindamycin-resistant *S. aureus* exceeds 15%, alternative antibiotics should be used as empiric therapy to improve outcomes [4,5,6,7,8,9]. *S. aureus* is a frequent colonizer in patients with AD; approximately 70% of affected individuals exhibited colonization in lesional skin, compared to 10% of healthy individuals [24,25]. Our understanding of this species’ role in AD has improved recently. Recent longitudinal analyses have shown that the skin microbiome of children with atopic dermatitis is dominated by *S. aureus*, and that its abundance decreases with treatment, gradually restoring a healthy microbial balance and correlating with clinical improvement [26], and the bacterium plays a role in epidermal barrier malfunction and inflammation [27,28]. Moreover, *S. aureus* evolves and adapts within hosts with AD and likely develops additional virulence factors that contribute to adhesion, inflammation, and barrier alterations, such as clumping factors and loss of capsule elements [16,29]. Specific clonal complexes have been found in patients with AD and are associated with disease exacerbation [3,27,30].

Biofilms produced by *S. aureus* protect it from antimicrobial peptides and phagocytosis, enabling persistence in the host [12]. In our study, we found that 60.2% of the isolates were moderate-to-strong biofilm producers, a finding similar to that of a previous study on children with AD [31]. One study reported that 67% of *S. aureus* strains were strong biofilm producers, whereas strains from healthy carriers exhibited lower biofilm-forming capacity [31]. These findings suggest a potential role for biofilms in the pathogenesis of AD [32]. Thus, the significant associations observed herein between increased biofilm production and baseline disease severity, lack of treatment response, and persistent moderate-severe AD throughout the study visits are relevant. Our results align with those of Di Domenico et al., who reported a correlation between biofilm formation and increased severity of AD lesions. Additionally, they noted that *S. aureus* biofilm production occurred in both the acute and chronic phases of the disease [33].

It is hypothesized that biofilms contribute to sweat duct occlusion and skin inflammation by inducing keratinocyte proliferation and cytokine secretion [31]. These processes may collectively impair skin barrier function, promote sensitization, and exacerbate pruritus, thereby prolonging the course of the disease [14]. However, biofilm production is also a successful strategy that protects bacteria from environmental threats and treatments such as antibiotics, potentially contributing to increased resistance of *S. aureus* to conventional antibiotics [14]. Importantly, all the isolates studied were methicillin-sensitive *S. aureus* (MSSA) strains. However, some studies have reported a low prevalence of MRSA in children with AD [34], along with an absence of multidrug resistance in isolates from both children and adults with AD [35]. MRSA strains are associated with more severe infections and exhibit greater antibiotic resistance complexity. However, it remains controversial whether MRSA strains are inherently more virulent than MSSA strains [36,37].

We found that more than 50% of the MSSA strains studied produced moderate to strong biofilms. A potential difference in biofilm formation between these two bacterial types has been suggested: a review of the differences in biofilm production between MRSA and MSSA was performed, and among the 20 research articles analyzed, 50% reported that MRSA isolates form biofilms better than MSSA, 45% reported no differences, and only 5% reported that MSSA formed biofilms better than MRSA. Future analyses should focus on better characterizing the isolates to provide more detailed information on their clonal origin [38]. The ability of strains to form biofilms, combined with their characteristic resistance profile, significantly contributes to an overall increase in bacterial resistance. Biofilms create a protective barrier that impedes the action of antibiotics, and the multidrug resistance profile further amplifies this challenge, severely limiting the availability of therapeutic options [39]. Consequently, this synergistic interaction between biofilm formation and multidrug resistance can lead to persistent therapeutic failure, highlighting the urgent need for innovative strategies to combat biofilm-associated infections. Our findings of suboptimal antimicrobial sensitivity to antibiotics commonly used to empirically treat AD flares and/or superinfections, such as CC and E, are relevant. A pivotal study by Allen et al. revealed, as did our findings, that the antibiotics with the highest percentages of isolates showing resistance were E (85.7%), CC (80.0%), and levofloxacin (65.7%) [31]. However, we obtained no MRSA isolates, unlike studies in other populations, where rates reached 60% [31]. In a recent meta-analysis, the antimicrobial susceptibility of *S. aureus* isolated from patients with AD to commonly prescribed beta-lactams, E, CC, and fusidic acid was found to be <85% [4], a proposed threshold at which an antibiotic should no longer be considered an empirical therapy [40]. All these findings emphasize that antibiotic treatment in patients with AD should be reserved for clinically evident superinfections and should always be guided by culture results.

We observed particular antimicrobial susceptibility profiles for the studied *S. aureus* isolates. In patients who did not have severe AD at baseline, in those whose severity improved throughout the study, and in those in whom the disease was not persistently severe, isolates were more frequently resistant to P. Strains of *S. aureus* resistant to P have been documented since 1942 [41]. The current resistance rates to this antibiotic range from 14% to 26% for MSSA (data from 1997 to 2016) [42], but in other studies, resistance rates as high as 85% have been reported [43,44]. Thus, we expected the isolates studied to show high resistance to P. Resistance to P does not necessarily imply increased virulence of *S. aureus*, but in some reports, the coexistence of P resistance in MSSA isolates and the presence of Panton-Valentine leukocidin has been reported [45,46]. Although this agent is rarely used to treat patients with AD and SSTIs, we decided to include it in testing, anticipating a low frequency of MRSA isolates in the cohort studied. Characterizing antimicrobial susceptibility to P provides relevant epidemiological information, indicating that the beta-lactam susceptibility profiles of AD patients differ from those of patients with other types of infection [42,47].

Patients who did not exhibit improvement harbored isolates more likely to be resistant to CIP, GM, CC, and E. High rates of E and CC resistance have been observed in MSSA, but a relationship with microbiological treatment has not been established [43,44,47]. Further studies could confirm or rule out this association. Data on MLS_B_ phenotypes in *S. aureus* from AD patients are limited. In this collection, the iMLS_B_ phenotype was observed in 14.7% of the isolates, and notably, all the isolates were MSSA. The distribution of the iMLS_B_ phenotype is reported to differ between MRSA and MSSA [18]; in MRSA, the frequency varies from 0 to 76.4% [48,49,50], whereas in MSSA, it ranges from 5.9 to 66% [49,51,52]. In China, Japan, and Brazil, the *erm*A and *erm*B genes are predominant among MRSA and MSSA [49,51,53]; however, in studies on isolates from other regions of Asia and Africa, *erm*C was the most frequently detected gene (40.7–72.4%) [48,54,55]. Information from Mexico about the genes involved in MLS_B_ phenotypes is limited; in a collection of 21 MRSA isolates obtained from catheter-associated infections, the coexistence of *erm*A and *erm*B was observed [56].

In this study, the *erm*A gene was detected in 80% of the isolates with the iMLS_B_ phenotype. No gene related to the iMLS_B_ phenotype was amplified from four isolates suggesting that another *erm* allele, such as *emr*T, may be involved, as it has been detected in isolates from the hospital environment and the nostrils of inpatients and health workers [55,57]. We found a significant association between the cMLS_B_ and iMLS_B_ phenotypes in isolates from patients who showed no improvement throughout the study, who had persistent moderate–severe AD, and who did not receive diluted bleach baths. These findings merit further study in prospective larger-sample studies. All these findings support the need to develop targeted treatments with mechanisms of action distinct from those of antibiotics to reduce the abundance of *S. aureus* and its virulence and pathogenic factor levels, particularly those related to biofilm production and quorum sensing.

Sodium hypochlorite, which is commonly used in the management of AD at concentrations ranging from 0.005 to 0.006% as an adjunctive treatment, has recently been shown in a meta-analysis to be effective in reducing AD severity and *S. aureus* abundance [58]. It has also proven effective against *S. aureus* biofilms. However, the concentrations required to eliminate *S. aureus* biofilms are greater than those for planktonic cells at concentrations between 0.01% and 0.08%. In vitro eradication of *S. aureus* biofilms was achieved at concentrations ranging from 0.01% to 0.16%, much higher than those currently used in bleach baths for patients with AD (0.006%) [59,60]. We found that adjunctive dilute bleach baths at follow-up visits were associated with resistance to CIP and constitutive and inducible MLS_B_. Although no studies support this association in *S. aureus*, previous studies on *Candida albicans* have reported that adaptation to sodium hypochlorite renders it ineffective [61].

We believe our findings are relevant and warrant further in vitro and in vivo studies to establish whether sodium hypochlorite may play a potential role in gene expression, protein transcription, adaptation, or other mechanisms of resistance in *S. aureus*. In terms of limitations, our results might be skewed by selection bias, as all the isolates were obtained from patients cared for at a tertiary institution who might have antimicrobial susceptibility profiles and biofilm production phenotypes. Additionally, we did not test for other relevant antibiotics or perform functional studies that might have shed light on *S. aureus* pathogenicity and virulence in the context of AD. However, data on the specific characteristics of *S. aureus* as a colonizer and pathogen in the skin of patients with AD are scarce. In Mexico, data on the antimicrobial susceptibility patterns of bacteria causing soft tissue and skin infections are scarce, especially for bacteria from the skin of patients with AD. Our findings contribute to epidemiological data on antimicrobial susceptibility profiles, are essential for establishing regional treatment guidelines, and offer further insights into the role of *S. aureus* in the pathophysiology of AD.

## 5. Conclusions

In conclusion, 60.2% of *S. aureus* isolates from children with AD were moderate or strong biofilm producers, and this was significantly associated with severe AD at baseline, a lack of response to treatment, and persistent moderate-severe AD throughout the study visits. We also found suboptimal resistance rates to frequently used and recommended empiric antibiotics in the context of AD, such as CC, P, and E, which could be a consequence of biofilm-mediated survival strategies, among other factors. Our findings indicate that adequate treatment of SSTIs in patients with AD should always be guided by culture and antibiograms. Moreover, these results highlight the need to study further host-microbial interactions and their implications for AD, as well as increase our understanding of staphylococcal biofilms in the context of AD, to facilitate the development of targeted treatments to reduce skin colonization, improve barrier function, weaken the immune response, and reduce flares while not promoting antibiotic resistance.

## Figures and Tables

**Table 1:** Clinical characteristics of 26 patients from whom *S. aureus* was isolated (n = 136).

Clinical Characteristics	Number of Isolates (%)
Site	50 (36.7)
Affected skin	
Unaffected skin	31 (22.7)
Nares	55 (40.4)
Severity of AD at baseline ^a^	
Moderate	42 (58.3)
Severe	30 (41.7)
Visit	
Baseline	72 (52.9)
Subsequent	64 (47)
Body sites colonized by *S. aureus* at baseline ^a^	
≤3	14 (19.4)
>3	58 (80.6)
Adjunctive treatment with dilute bleach baths ^b^	
No	41 (62.1)
Yes	23 (37.9)
SCORAD decreased 15 or more points from baseline to 4th visit	
No	40 (29.4)
Yes	96 (70)
Persistently moderate-severe AD	
No	78 (57.3)
Yes	58 (42.7)

Abbreviations: SCORAD: Severity score for AD; AD: atopic dermatitis. ^a^ Only samples from the baseline visit were analyzed (n = 72). ^b^ Only samples from subsequent visits were analyzed (n = 66).

**Table 2:** Patterns of susceptibility/resistance to the tested antibiotics in all the *S. aureus* isolates (n = 136).

Variables	LZ	CC	GM	CIP	P	E	TE	SXT	FOX
Resistant, n (%)	0	34 (25)	87 (64)	1 (0.7)	103 (75.7)	34 (25)	3 (2.2)	1 (0.7)	0
Intermediate, n (%)	–	–	–	21 (15.4)	–	1 (0.7)	9 (6.6)	0	–
Sensitive, n (%)	136 (100)	102 (75)	49 (36)	114 (83.8)	33 (24.3)	101 (74.3)	124 (91.2)	135 (99.3)	136 (100)

Abbreviations: LZ: linezolid; CC: clindamicin; GM: gentamicin; CIP: ciprofloxacin; P: penicillin; E: erythromycin; TE: tetracycline; FOX: cefoxitin (proxy for methicillin resistance); SXT: trimethoprim-sulfamethoxazole.

**Table 3:** *S. aureus* MLS_B_ sensitivity (n = 136).

Variables	Number of Isolates	Moderate-Strong Biofilm (n = 82)	*p* Value
	n (%)	n (%)	
E-S, CC-S	101 (74.2)	62 (61.4)	NS
E-R, CC-R (constitutive MLS_B_)	14 (10.2)	8 (57.1)	NS
E-R, CC-S (D-test positive, inducible MLS_B_)	20 (14.7)	11 (55)	NS
E-R, CC-S (D-test negative, MS)	1 (0.7)	1 (100)	NS
Indeterminate	0	0	NS

E: erythromycin; S: sensitive; R: resistant; CC: clindamycin; MLS_B_: macrolide-lincosamide-streptogramin B resistance; MS: Macrolide-Streptogramin B. Not significant (NS).

**Table 4:** Antibiotic resistance in *S. aureus* isolates and clinical characteristics of the studied population.

	Intermediate/Resistant								Biofilm Production
	CC, n (%)	GM, n (%)	CIP, n (%)	P, n (%)	E, n (%)	TE, n (%)	SXT, n (%)	FOX	Moderate/Strong, n (%)
All isolates	34	49	22	103	35	12	1	0	82
Site									
Nonlesional (n = 50)	11 (32.4)	19 (38.8)	10 (45.4)	37 (35.9)	11 (31.4)	6 (50)	0		29 (35.4)
Lesional (n = 31)	8 (23.5)	9 (18.4)	4 (18.1)	22 (21.4)	8 (22.8)	3 (25)	1 (100)		22 (26.8)
Nares (n = 55)	15 (44.1)	21 (42.9)	8 (36.3)	44 (42.7)	16 (45.7)	3 (25)	0		31 (37.8)
Type of visit									
Baseline visit (n = 72)	14 (41.2)	25 (51)	8 (36.3)	55 (53.4)	14 (40)	6 (50)	1 (100)		38 (46.3)
Follow-up visits (n = 64)	20 (58.8)	24 (49)	14 (63.7)	48 (46.6)	21 (60)	6 (50)	0		44 (53.7)
SCORAD decreased ≥ 15 from baseline to final visit									
Yes (n = 40)	16 (47.1)	20 (40.8)	16 (72.7)	22 (21.4)	16 (45.7)	4 (33.3)	0		29 (35.4)
No (n = 96)	18 (52.9) **	29 (59.2) *	6 (27.3) ***	81 (78.6) ***	19 (54.3) *	8 (66.6)	1 (100)		53 64.6) *
Persistently moderate-severe AD									
Yes (n = 58)	25 (73.5)	41 (83.7)	16 (72.7)	35 (34)	26 (74.2) ***	2 (16.6) *	0		42 (51.2)
No (n = 78)	9 (26.5) ***	8 (16.3) ***	6 (27.3) **	68 (66) ***	9 (25.8)	10 (83.4)	1 (100)		40 (48.8) *
Isolates from baseline visit only, (n = 72)	14	25	8	55	14	6	1	0	38
No. of sites colonized by *S. aureus* at baseline									
<3 sites ^a^ (n = 14)	1 (7.1)	4 (16)	0	12 (21.8)	1 (7.1)	1 (16.7)	0		5 (13.2)
≥3 sites ^a^ (n = 58)	13 (92.9)	21 (84)	8 (100)	43 (78.2)	13 (92.9)	5 (83.3)	1 (100)		33 (86.8)
Severity of AD at baseline									
Nonsevere AD ^a^ (n = 42)	7 (50)	14 (56)	5 (62.5)	36 (65.5)	7 (50)	4 (66.6)	0		7 (44.7)
Severe ^a^ (n = 30)	7 (50)	11 (44)	3 (37.5)	19 (34.5) *	7 (50)	2 (33.3)	1 (100)		21 (55.3) *
Isolates from subsequent visits only, (n = 64)	20	24	14	48	21	6	0	0	44
Type of treatment									
Adjunctive diluted bleach baths ^b^ (n = 23)	7 (35)	10 (41.7)	1 (7.2) *	22 (45.8) **	8 (38.1)	1 (16.6)	0		19 (56.8)
Standard treatment ^b^ (n = 41)	13 (65)	14 (58.3)	13 (92.8)	26 (54.2)	13 (61.9)	5 (83.4)	0		19 (43.2)

Notes: SCORAD: Severity score for AD; AD: atopic dermatitis; LZ: linezolid; CC: clindamicin; GM: gentamicin; CIP: ciprofloxacin; P: penicillin; E: erythromycin; TE: tetracycline; FOX: cefoxitin; SXT: trimethoprim-sulfamethoxazole. * *p* = 0.05; ** *p* < 0.001; *** *p* < 0.0001. ^a^ Only samples from the baseline visit were analyzed (n = 72); ^b^ Only samples from subsequent visits were analyzed (n = 64).

## Data Availability

The original contributions presented in this study are included in the article/Supplementary Materials. Further inquiries can be directed to the corresponding author.

## Supplementary Materials

The following supporting information can be downloaded at: https://www.mdpi.com/article/10.3390/microorganisms14020311/s1, Table S1. Antimicrobial susceptibility of *Staphylococcus aureus* isolates: inhibition zone diameter ranges and breakpoint (CLSI, 2023). Table S2. Primer sequences used for the detection of *erm*A, *erm*B, *erm*C, and *msr*A genes in *Staphylococcus aureus* isolates. Table S3. Mean OD₅₇₀ values (± SD) according to biofilm production categories. Figure S1. Representative agarose gel electrophoresis showing PCR amplification of MLSB-related genes in any *Staphylococcus aureus* isolates and positive controls: *erm*A (lines samples 1, 2 positive control 3 and no-template control, NTC 4); *erm*B (lines samples 5, 6 and 7); *erm*C (lines samples 8, 9, 11, Non-study isolate 10, positive control 12 and no-template control NTC 13) Molecular weight marker, M.

## Abbreviations

AMPs	Antimicrobial peptides
cMLS_B_	constitutive macrolide-lincosamide-streptogramin B resistance
iMLS_B_	inducible macrolide-lincosamide-streptogramin B resistance
ICR	Inducible clindamycin resistance
MRSA	Methicillin-resistant *Staphylococcus aureus*
MSSA	Methicillin-sensitive *Staphylococcus aureus*
AD	Atopic dermatitis
LZ	Linezolid
CC	Clindamycin
GM	Gentamicin
CIP	Ciprofloxacin
P	Penicillin
E	Erythromycin
TE	Tetracycline
FOX	Cefoxitin
SXT	Trimethoprim-sulfamethoxazole
