# Supplementary material for: Antimicrobial Susceptibility Patterns and Biofilm Formation of Staphylococcus aureus Strains Isolated from Pediatric Patients with Atopic Dermatitis
Source: Microorganisms. 2026 Jan 29;14(2):311. doi: 10.3390/microorganisms14020311 (PMC12943069; doi:10.3390/microorganisms14020311)
Supplement: Supplementary file 1 [file microorganisms-14-00311-s001.zip › microorganisms-3964175-supplementary.pdf]

Supplementary material

Supplementary Table S1. Antimicrobial susceptibility of *Staphylococcus aureus* isolates: inhibition zone diameter ranges and breakpoint (CLSI, 2023).

Antibiotic	CLSI breakpoint (mm)	Range of inhibition zones (mm)	Mean ± SD
Penicillin (P, 10 U)	≤28	11–43	21.77 ± 9.91
Cefoxitin (FOX, 30 µg)	≤21	22–31	27.03 ± 1.62
Erythromycin (E, 15 µg)	≤13	6–29	21.21 ± 8.75
Clindamycin (CC, 2 µg)	≤14	6-29	20.39 ± 8.39
Ciprofloxacin (CIP, 5 µg)	≤15	15-35	26.32 ± 4.49
Gentamicin (GM, 10 µg)	≤12	6-24	17.38 ± 7.09
Linezolid (LZD, 30 µg)	≤20	26-34	28.97± 1.73
Tetracycline (TE, 30 µg)	≤14	0-38	24.60 ± 5.53
Trimethoprim-sulfamethoxazole (SXT, 30 µg)	≤10	0-31	25.57 ±3.28

Supplementary Table S2. Primer sequences used for the detection of *ermA*, *ermB*, *ermC*, and *msrA* genes in *Staphylococcus aureus* isolates.

Gene	Amplicon size (bp)	Forward primer (5′–3′)	Reverse primer (5′–3′)	Amplification conditions
ermA	139	TATCTTATCGTTGAGAAGGGATT	CTACACTTGGCTTAGGATGAAA	1. Denaturation (95°C for 60 sec)<br><br>2. Denaturation for 94°C for 45 sec, annealing 55°C for 30 sec and extension 72°C for 60 sec for33 cycles.<br><br>3. Final extension phase (72°C for 5 min)
ermB	359	CCGTTTACGAAATTGGAACAGGTAAAGGGC	GAATCGAGACTTGAGTGTGC	
ermC	295	ATCTTTGAAATCGGCTCAGG	CAAACCCGTATTCCACGATT	
msrA	163	TCCAATCATTGCACAAAATC	AATTCCCTCTATTTGGTGGT	

Positive controls: *Staphylococcus aureus* strains O2 (*erm*A and *erm*C) and O46 (*erm*A), previously characterized and sequenced by our group [23].
The primers used were designed according to Khodabandeh et al., [22]

Supplementary Table S3. Mean OD_{570} values (± SD) according to biofilm production categories

Category	n	Mean OD_{570} ± SD
Non-biofilm-formers (NBF)	23	0.154 ± 0.028
Weak biofilm-formers (WBF)	31	0.255 ± 0.035
Moderate biofilm-formers (MBF)	55	0.451 ± 0.086
Strong biofilm-formers (SBF)	27	0.811 ± 0.324

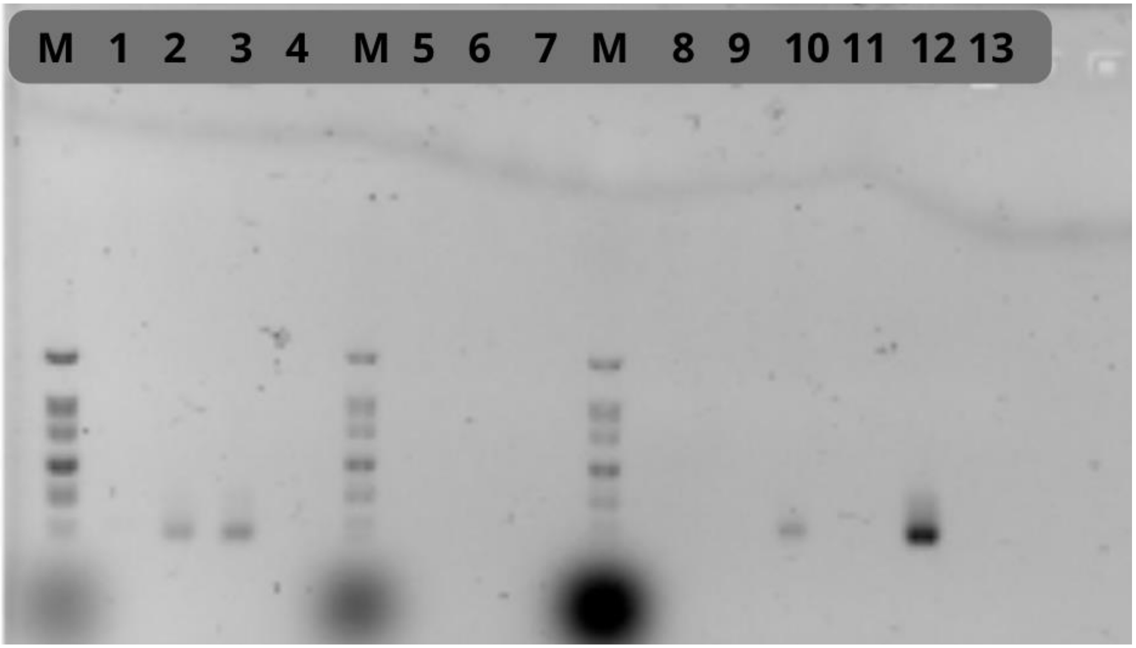

Figure S1. Representative agarose gel electrophoresis showing PCR amplification of MLSB-related genes in any *Staphylococcus aureus* isolates and positive controls: *erm*A (lines samples *1, 2* positive control *3* and no-template control, NTC *4*); *erm*B (lines samples *5, 6* and *7*); *ermC* (lines samples *8,9,11*, Non-study isolate *10*, positive control *12* and no-template control NTC *13*) Molecular weight marker, M.
